# Type 2 immunity in intestinal homeostasis and inflammatory bowel disease

**DOI:** 10.1042/BST20210535

**Published:** 2021-09-28

**Authors:** Xinxin Luo, Eduardo J. Villablanca

**Affiliations:** 1 Division of Immunology and Allergy, Department of Medicine Solna, Karolinska Institute and University Hospital, Stockholm, Sweden; 2 Center of Molecular Medicine, 17176 Stockholm, Sweden

**Keywords:** ILC2, inflammatory bowel disease, mucosal immunology, T_H_2, type 2 immunity

## Abstract

Type 2 immune responses commonly emerge during allergic reactions or infections with helminth parasites. Most of the cytokines associated with type 2 immune responses are IL-4, IL-5, and IL13, which are mainly produced by T helper 2 cells (T_H_2), eosinophils, basophils, mast cells, and group 2 innate lymphoid cells (ILC2s). Over the course of evolution, humans have developed type 2 immune responses to fight infections and to protect tissues from the potential collateral damage caused by inflammation. For example, worm parasites induce potent type 2 immune responses, which are needed to simultaneously clear the pathogen and to promote tissue repair following injury. Due to the strong type 2 immune responses induced by helminths, which can promote tissue repair in the damaged epithelium, their use has been suggested as a possible treatment for inflammatory bowel disease (IBD); however, the role of type 2 immune responses in the initiation and progression of IBD is not fully understood. In this review, we discuss the molecular and cellular mechanisms that regulate type 2 immune responses during intestinal homeostasis, and we briefly discuss the scarce evidence linking type 2 immune responses with the aetiology of IBD.

## Introduction

**Figure 1.**
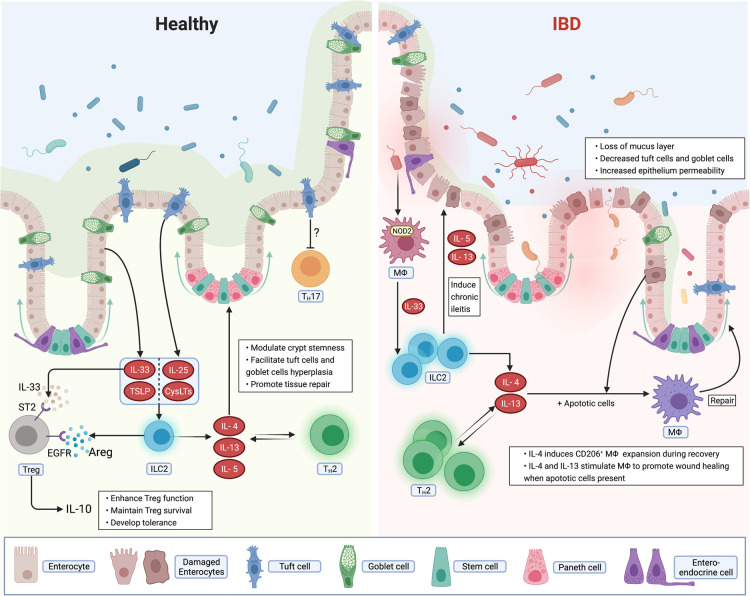
Type 2 immune responses in the intestinal mucosa from healthy and inflammatory bowel disease (IBD). Type 2 immune responses mediated by T_H_2 and ILC2 during homoeostatic conditions, inflammation and tissue repair: (**a**) At steady-state conditions, type 2 cytokines produced by either ILC2 or T_H_2 orchestrate epithelial homeostasis. Type 2 immune responses can promote crypt stemness and the epithelial cell differentiation towards the tuft and/or goblet cell lineage. This process is crucial to maintain a healthy mucus layer and eventually the integrity of the intestinal barrier. Epithelial cells derived alarmins (IL-25, CysLTs, IL-33, and TSLP) activate ILC2 promoting the establishment of tolerogenic immune responses. (**b**) In the inflamed intestine during IBD, ILC2, and T_H_2 accumulate in inflamed lesions. Damaged epithelium permeability and loss of the mucus layer might result in commensals bacterial translocation and dissemination. Dissemination of bacteria result in NOD2-dependent activation which results in the production of IL-33. Eventually, ILC2s are activated by IL-33 and in turn produce IL-5 and IL-13, which fuel chronic ileitis. (**c**) During the process of tissue repair, IL-4 or IL-13 combined with apoptotic cells stimulate macrophages to promote wound healing and tissue remodeling.

The breakdown of intestinal homeostasis may lead to aberrant immune responses against luminal antigens and eventually lead to inflammatory bowel diseases (IBD), which includes Crohn's disease (CD) and ulcerative colitis (UC). Although the aetiology of IBD is not well understood, it is broadly accepted that genetic and environmental factors are key [1,2]. The incidence of IBD in developed countries and regions with superior hygiene standards is higher than in regions with notable parasite exposure [3]. The removal of these co-evolved parasites may alter the establishment of tolerogenic and immunoregulatory responses [3]. Thus, parasite exposure may benefit the immune system by conferring protection against IBD. Parasite infection also known as helminths infection such as intestinal residing hookworm [4] promotes strong type 2 immune responses, wherein T helper 2 cells (T_H_2) and innate lymphoid cells class 2 (ILC2s) are the major drivers of such responses [5,6]. Type 2 immune responses play an important role in epithelial barrier tissues. The epithelium, specifically tuft cells [7], control type 2 immune responses, highlighting a bi-directional epithelium-immune circuit orchestrated by type 2 cytokines. In this mini-review, we discuss and summarize the recent evidence linking type 2 immune responses and intestinal homeostasis, as well as how the failure of this regulating pathway may lead to IBD (Figure 1).

## Immune cells in type 2 immune responses and IBD

IBD is characterized by activation of T cell-derived pro-inflammatory cytokines, such as IL-17 [8], TNFα [9], and IFN-γ [10,11], as well as malfunction of peripheral- and tissue-specific regulatory T cells [12,13]. T_H_1 and T_H_ 17 immune responses, dominated by the production of IL-12, IL-23, and IL-17 cytokines, have been well-studied in IBD [8]. However, the role of T_H_2 immunity in IBD is not well understood. Here we discuss the potential role of T_H_2 and its innate counterpart ILC2 in the aetiology of IBD.

### T_H_2 in IBD

T_H_2 immune responses contribute to tissue repair and damage control, which can be considered as the counterpart to T_H_1 immune responses [14]. The activation of transcription factors associated with T_H_2 differentiation (e.g. GATA3, STAT6, and c-MAF), represses T_H_1 or T_H_17 differentiation, thus inhibiting the production of type 1 and type 17 effector cytokines (e.g. IFNγ, IL-1β, TNFα, and IL-17), which are known to drive IBD pathogenicity [15–17].

Although direct links between T_H_2 immune response and IBD have not been shown, there is emerging research showing indirect links. T_H_2 cells are more abundant in the lamina propria of UC compared with CD patients [2]. Moreover, T_H_2 cells are enriched in CD patients who do not respond to anti-TNF therapy, suggesting that increased T_H_2 is associated with IBD severity [18]. Despite the accumulation of T_H_2 cells in inflamed tissues, it is not known whether T_H_2 cells are beneficial or detrimental. However, it is known that the type 2 cytokine IL-4 promotes macrophage-mediated wound healing and alleviates colitis [14,19]. A similar effect has been observed in studies with IL-13, which together with apoptotic cells, is required in macrophage-dependent pathogen clearance and tissue integrity restoration [20]. Phenome-wide association studies also found that carriers of the R130Q variant links high IL-13 activity with low susceptibility for developing CD [21]. Moreover, sustained CD4T cell-derived IL-13 activity protects mice from experimental colitis [21]. A recent case report suggested that blocking IL-4 activity using the IL-4Ralpha antagonist dupilumab may cause enteritis as a side effect when initially used to treat atopic dermatitis [22]. Thus, T_H_2 immune response may play a protective role in the pathogenesis of IBD.

On the other hand, T_H_2 cells may directly cause intestinal inflammation, as seen by experiments in which IL-4 expressing CD4^+^ T cells from mice with ileitis were sufficient to initiate ileitis upon being adoptively transferred in immunocompromised recipients [23]. In addition, despite the primarily role of T_H_1 immune responses in the establishment of ileitis in SAMP1/YitFc mice, the terminal ileal tissue manifested an elevated type 2 immunity signature (IL-5, IL-13, and GATA3/Tbet ratio) during chronic inflammation compared with the healthy control [23].T_H_2 responses are also the driving force in an oxazolone-induced colitis murine model, which can be attenuated by suppressing type 2 cytokines [24–26]. Blocking GATA3 with a specific DNAzyme reduces inflammation histologically in a 2,4,6-trinitrobenzene sulfonic acid (TNBS)-induced colitis murine model [27]. In human, UC patients show enhanced type 2 response in their mucosal samples compared with CD [18]. Interestingly, the source of IL-13 may be type 2 natural killer T cells rather than conventional T_H_2 cells [28,29]. Contrasting with the preclinical data, the IL-13 neutralizers tralokinumab and anrukinzumab did not achieve clinically significant responses in UC patients [30,31]. These contradictory findings may reflect the heterogeneous aetiology and immunological response in IBD patients. Future studies might address the source of type 2 cytokines and how their interplay with T_H_2 cells contributes to the disease development.

### Type 2 innate lymphoid cells (ILC2) in IBD

Unlike T_H_2, which responds to antigen-specific activation signals, ILC2 responds to a broader range of stimuli derived from immune cells, epithelial cells, or even neurons [32] and, functions in parasite invasion, allergic reactions, tissue repair, and intestinal homeostasis [33]. ILC2 serves as an initial response before the adaptive immune system resolves the challenge. When challenged, the tuft cell-derived IL-25 activates small intestine resident ILC2s. ILC2 attracts and activates T_H_2 cells by producing the type 2 cytokines IL-4, IL-5, and IL-13 [7,34]. As a result, effector cytokines such as IL-13 promote tissue remodeling by modulating crypt stemness and stimulating goblet cell (GC) and tuft cell hyperplasia in order to facilitate pathogen clearance [34–36].

Besides acting on epithelial cells, ILC2 impacts other immune cells. For example, ILC2 produces and secretes amphiregulin (AREG), which can trigger epidermal growth factor receptor (EGFR) signaling of regulatory T cells (Treg) to consequently enhance their immunosuppressive potential [37–39]. Moreover, similar to negative selection in the thymus, ILC expresses MHC II molecules, which deplete commensal bacteria-specific CD4+ T cells [40]. Although this mechanism has been fully demonstrated in ILC3s, studies have demonstrated that MHC II is also expressed on ILC2s [41,42]. Emerging research also highlights the dialog between ILC2 and the enteric nervous system (ENS), which is crucial for establishing homeostasis. Neuromedin U (NMU), a neuropeptide produced by neurons, can be sensed by NMU receptor 1 (NMUR1)-expressing ILC2s, which are restricted to the gut [43]. NMUR1 signaling is sufficient to activate and induce ILC2-derived type 2 immune responses [43]. This neuron-ILC2 axis seems to be relevant to oral tolerance as the immune-suppressive cytokine IL-10 is highly regulated by the NMU-NMUR1 axis [44]. In addition, choline acetyltransferase (ChAT+) enteric neurons targets ILC2 through the production of the α-calcitonin gene-related peptide, which inhibits ILC2-induced inflammation and tuft cell expansion [45]. Thus, the cross-talk between ENS and ILC2 may be critical for establishing intestinal immune homeostasis.

In patients diagnosed early with IBD, increased ILC2 (restricted to inflamed tissue), was observed in UC, but not in CD patients, when compared with non-inflamed or healthy specimens [46]. However, such observations were no longer seen in UC and CD patients with at least a 1-year medical history [46]. This observation is in agreement with a recent study showing ILC2 accumulation in CD patients with a dysregulated bacteria-sensing processes [47]. Regardless whether ILC2 plays a protective or pathogenic role in IBD [48,49], the relationship between ILC2, the innate mucosal barrier, as well as immune and neural modulation, provides new therapeutic targets for IBD treatment.

## Intestinal epithelial cells

The intestinal barrier is composed primarily of a single layer of epithelial cells, which produce biochemical molecules that reinforce the physical barrier. The intestinal barrier acts as the first line of defence against possible pathogens; its malfunction may result in pathogen translocation into the lamina propria, triggering a cascade of events leading to IBD [50]. Many intestinal barrier components, such as tuft cells and mucin-producing GCs, are regulated by type 2 immunity.

### Tuft cell

Intestinal tuft cells are chemoreceptive cells characterized by the presence of brush-like microvilli projecting to the intestinal lumen. Although tuft cells were first discovered in the 1950s, its critical role in maintaining intestinal homeostasis has only been recently characterized. This knowledge gap is likely due to limited research tools to visualize and investigate tuft cells who are low in abundance both in experimental models and human (e.g. tuft cells only make up 0.4% of the cells in the mouse intestinal epithelium) [51,52]. Recent studies have shown that tuft cells sense luminal cues, such as microbe-derived metabolites [53] and, regulate the invasion of pathogens, such as protists [54], helminth [55], and/or virus [56]. In response to pathogen invasion, tuft cells significantly expand and secrete large amounts of IL-25 and cysteinyl leukotrienes (cysLTs), which subsequently activate ILC2 [54]. In turn, tuft cell expansion required IL-13 and IL-5 signals given the evidence that deletion of these two genes impaired tuft cell hyperplasia in response to *Nippostrongylus brasiliensis* [34]. In the TNFα overexpressing mouse model of colitis, administration of succinate resulted in tuft cell hyperplasia and decreased ileum inflammation [57]. In addition to tuft cell hyperplasia, treated mice demonstrated lower type 17 cytokines and retinoic acid receptor-related orphan receptor gamma-t (RORγt+) cells compared with control mice [57]. Banerjee and colleagues reported that CD patients possess tuft cell deficiency in ileal lesions [57]. Tuft cell-associated IBD phenotypes is not restricted to the ileum; a pediatric study reported that patients with acute duodenitis, ulcer, or celiac disease showed significantly lower tuft cells in the duodenum compared with controls.

Inflammation negatively impacts tuft cells. A previous study showed that inflammatory severity inversely correlates with tuft cell count [58]. In mouse, doublecortin like kinase 1 (DCLK1) is a broadly used marker for tuft cell analysis. Conditional knock out mice lacking DCLK1 in intra epithelial cells develops spontaneous colitis from age around 3–4 weeks and this was associated with decreased COX-2 expression and therefore the reduced prostaglandin E2 (PGE2) production, which is a key regulator in colonic epithelial cells proliferation [59]. *Dclk1*^−/−^ mice also showed delayed tissue regeneration following intestinal damage [60]. In agreement with a potential role in tissue regeneration, DCLK1 deficiency reduces the development of intestinal adenomas and limits pro-survival signaling and self-renewal in *Apc*^Min/+^ mice [61,62]. The mechanism by which tuft cells orchestrates tissue remodeling through cross-talk with immune cells requires future investigation. Moreover, whether the reduction in tuft cells in IBD lesions is a protective response or the consequence of IBD remains to be fully understood.

### Goblet cells

GCs are epithelial cells with a narrow basal end and wide apical surface. GCs in the small intestine and colon share the same functional property, which is to secrete the mucus-producing mucin. As the main function of the mucus layer is to lubricate the intestinal lumen and to protect it from pathogen invasion, GCs are critical for maintaining the barrier integrity. In agreement, mucin-2 (*Muc2*)-deficient mice show spontaneous colitis [63]. GC hyperplasia is often seen in allergies or helminth infections, wherein the T_H_2 immune response is enhanced. ILC2 has been proposed to be a major modulator of GC homeostasis and function [7,34,55]. In experimental colitis, IL-33 induced ILC2 activation, resulting in GC hyperplasia that contributes to epithelial structural restoration [64]. In addition, ILC2-derived IL-13 induces GC hyperplasia in an *in vitro* organoid culture system [65]. However, whether T_H_2 and/or ILC2 have direct effects on GC homeostasis and function in IBD patients needs to be determined.

Aberrant GC function has been characterized in UC patients. A proteomic study in active UC patients demonstrated that MUC2 was significantly decreased in both colonic lesions and uninflamed sites [66]. This phenotype is in line with a systematic review showing that 5 out of 8 human studies during the past 25 years reported decreased MUC2 protein levels in UC patients compared with healthy individuals [67]. The pathogenicity associated with decreased MUC2 was linked to increased bacterial penetration in the inner layer of mucus, which may lead to intestinal inflammation and dysbiosis [68]. Single-cell transcriptional analysis identified inter-crypt goblet cells (icGCs), a functionally distinct GC subpopulation from the canonical crypt-resident GCs [69]. Mucus secreted by icGCs seals the gap between opening crypts, thus preventing microbial penetration into the stem cells niche [69]. Moreover, UC patients demonstrated reduced icGCs and mucus defects [69]. In line with its protective role, icGC-deficient mice develop age-dependent colitis [69]. Whether icGC homeostasis and function is regulated by type 2 immunity, similar to conventional GCs, requires further investigation.

## Epithelium-derived type 2 immunity associated cytokines

Cytokines, which modulate intestinal barrier function, are crucial for the development of type 2 immune responses. In the following section, we will discuss classical epithelium-derived cytokines (called intestinal alarmins) that modulate type 2 immunity.

### IL-25

IL-25 (also named IL-17E) belongs to the IL-17 family and triggers the heterodimer complex IL-17RA and IL-17RB (IL-25R). IL-25, which is primarily produced and secreted by tuft cells, promotes type 2 responses [70]. The proliferation of IL-25 producing tuft cells is stimulated by IL-4, IL-5, and IL-13 in a positive feedback loop [34,70].

The *IL-25* gene is located within a region reported to confer susceptibility to IBD; however, a study in a small cohort of patients did not show the association between polymorphisms within the *IL-25* and IBD [71]. Studies in larger cohorts of IBD patients are needed to investigate the involvement of polymorphisms in the *IL-25* gene in IBD aetiology.

IL-25 can act on IL25R-expressing ILC2s, CD4^+^ T cells and mesenchymal stem cells (MSC) [72]. IL-25R^+^ CD4^+^ T cells and IL-25R^+^ Lgr5^+^ MSCs are enriched in the inflamed colonic mucosa of CD and UC patients compared with healthy individuals or non-inflamed tissue from IBD patients [72]. However, reductions in circulating IL-25R^+^ CD4^+^ was negatively correlated with inflammation-associated severity [72]. *In vitro* studies showed that IL-25 may prime Lgr5^+^ cells to secret pro-survival factors involved in the PI3K-Akt pathway to maintain epithelial cells homeostasis [72]. IL-25 was also found to ameliorate experimental TNBS- and dextran sodium sulfate (DSS)-induced colitis by inhibiting T_H_17 cells [72–75]. In line with these observations, Caruso and colleagues showed lower IL-25 transcriptional and protein levels in IBD colonic biopsies compared with healthy individuals [76]. Interestingly, IL-25R is expressed by CD14^+^ cells, a critical source of IL-12 in CD mucosa [76]. *In vitro* studies demonstrated that IL-25 signaling in CD14^+^ cells suppresses IBD inflammatory cytokines, such as IL-12 and IL-23 [76]. Furthermore, IL-25 treatment could reduce partially the TNBS-colitis histologic scoring, suggesting that IL-25 may limit inflammation development by reducing IL-12 and IFNγ protein expression and cellular infiltration to the mucosa [76]. IL-25 also shows tumor suppressive properties; blocking IL-25 signaling in a colitis-induced tumor model increases tumor numbers when compared with control mice [77].

Despite the research described above, some studies suggest that IL-25 may aggravate mucosal inflammation. Reynolds et al. [78] demonstrated that IL-25-deficient mice were protected from DSS-induced colitis; however, IL-25-deficient mice showed poorer outcomes compared with wild type control when infection was induced by *Citrobacter rodentium*. Thus, the role of IL-25 in either ameliorating or exacerbating intestinal inflammation may be context dependent.

### IL-33

The type 2 cytokine IL-33 belongs to the IL-1 family. In response to intestinal damage or stress, IL-33 is released by necrotic epithelial cells, stromal cells and/or endothelial cells in both mouse and humans [79]. IL-33, a potent driver of T_H_2 cells and ILC2 differentiation and function, plays an important role in allergic inflammation, which depends on IL-33 binding to its receptor ST2 (IL1RL1) and IL-1 receptor accessory protein (IL1RAP) [80].

The dual-function of IL-33 has led to several debatable insights. Recently, IL-33 has been reported to delay *C. rodentium* clearance by limiting T_H_17 responses and increasing intestinal permeability [81]. The pathogenic role of IL-33 was also observed in the SAMP1/YitFc (SAMP) mouse model of human CD ileitis [47]. In this study, the IL-33/ST2 pathway promoted NOD2-induced ILC2 expansion in ileitis; furthermore, blocking IL-33 protected SAMP mice [47]. Similar observations were seen in IL-33 deficient mice with DSS-induced colitis [48]. In inflamed colonic biopsies from UC patients, IL-33 was increased compared with inflamed or non-inflamed tissue samples from CD patients, indicating a pathogenic role in UC [82,83]. In contrast with the pathogenic role of IL-33, the protective role of IL-33 may involve the interaction with regulatory T cells. ST2 is one of the top differentially expressed genes in colonic Treg cells (cTreg) compared with Treg cells in mesenteric lymph node (MLN) [84]. Interestingly, cTreg differentiation and function is modulated by IL-33-ST2 signaling [84]. During inflammation, *Rag1*^−/−^ mice receiving the combination of CD45.1^+^ naïve T cells (RB^hi^) and ST2^−/−^ Treg have been reported to show impaired cTreg compared with RB^hi^ + wild type Treg, thus leading to more severe colitis [84]. Furthermore, IL-33 treatment reduced TNBS-colitis severity [85]. IL-33 can also favor tissue remodeling and induce tuft and GC function [64,65]. The failure to regulate the IL-33-tuft cell axis may be associated with SPRY2 (Sprouty2, an intracellular signaling regulator). *SPRY2* expression has been shown to be enhanced in both CD and UC patients, resulting in tuft cells and GC inhibition [35]. Collectively, different studies showing both pathogenic and protective roles of IL-33 suggest that IL-33 may be a double-edged sword.

### Thymic stromal lymphopoietin (TSLP)

TSLP, which is mainly secreted by epithelial cells and stroma cells [86], is regarded as a potent ILC2 activator. Its role has been extensively studied in dermic allergic and asthma [87]. The role of TSLP in UC patients remains controversial. Tanaka et al. found that TSLP was increased in inflamed lesions compared with non-inflamed tissues from UC patients. Furthermore, they showed that IL-4 and TNF-α may exacerbate TSLP expression, leading to enhanced inflammation [88]. However, others showed that TSLP was reduced in UC patients compared with healthy or non-inflamed tissues [89,90]. Patients receiving anti-IL-13 (tralokinumab) also showed more TSLP transcriptional expression with improved tissue healing compared before treatment [89]. TSLP may trigger ILC2 to produce AREG*,* which in turn promotes epithelium repair via the AREG/EGFR signaling pathway. In line with this observation, TSLP receptor-deficient mice showed poorer outcomes compared with their wild type control in DSS model [91]. Furthermore, exogenous delivery of TSLP using an engineered lactic acid bacteria ameliorated DSS-induced inflammation by inducing TGF-β, thus reinforcing its immunosuppressive function [92].

## Perspectives

- Type 2 immune responses are critical to control pathogen infections and to promote tissue repair whereas breakdown on these mechanisms may lead to IBD (Figure 1)
- The current literature linking type 2 immune responses and IBD is rather inconclusive, as pathogenic and protective roles of type 2 immune responses in IBD has been reported.
- More intensive research on the specific function of type 2 cytokines in specific phases of the disease are urgently needed to better interpret the role of type 2 immune responses in IBD.

## Open Access

Open access for this article was enabled by the participation of Karolinska Institute in an all-inclusive *Read & Publish* pilot with Portland Press and the Biochemical Society.

## Abbreviations

AREG: amphiregulin
CD: Crohn's disease
DCLK1: doublecortin like kinase 1
DSS: dextran sodium sulfate
EGFR: epidermal growth factor receptor
ENS: enteric nervous system
GCs: goblet cells
IBD: inflammatory bowel diseases
icGCs: inter-crypt goblet cells
MSC: mesenchymal stem cells
NMU: neuromedin U
NMUR1: NMU receptor 1
RORγt+: receptor-related orphan receptor gamma-t
T_H_2: T helper 2 cells
TNBS: 2,4,6-trinitrobenzene sulfonic acid
Treg: regulatory T cells
TSLP: thymic stromal lymphopoietin
UC: ulcerative colitis
